# Precautionary Behavior and Depression in Older Adults during the COVID-19 Pandemic: An Online Cross-Sectional Study in Hubei, China

**DOI:** 10.3390/ijerph18041853

**Published:** 2021-02-14

**Authors:** Wei Liang, Yanping Duan, Borui Shang, Chun Hu, Julien Steven Baker, Zhihua Lin, Jiali He, Yanping Wang

**Affiliations:** 1 Center for Health and Exercise Science Research, Hong Kong Baptist University, Hong Kong, China; wliang1020@hkbu.edu.hk (W.L.); jsbaker@hkbu.edu.hk (J.S.B.); 2 Department of Sport, Physical Education and Health, Hong Kong Baptist University, Hong Kong, China; 3 Wuhan Institute of Physical Education, College of Health Sciences, Wuhan 430000, China; h13163386915@163.com; 4 Department of Kinesiology, Hebei Institute of Physical Education, Shijiazhuang 050000, China; boruishang@hepec.edu.cn; 5 Student Mental Health Education Center, Northwestern Polytechnical University, Xi’an 710000, China; huchun52@163.com; 6 Sport Section, Wuhan University, Wuhan 430000, China; zhihualin_wu@163.com; 7 The National Physical Fitness Lab, Hubei Institute of Sport Sciences, Wuhan 430000, China; yanniswyp@163.com

**Keywords:** COVID-19, older adults, depression, precautionary behavior, socioeconomic status, online survey, mental health

## Abstract

The large-scale COVID-19 pandemic has not only resulted in the risk of death but also augmented the levels of depression in community-dwelling older adults. The present study aimed to investigate the characteristics of depression in Chinese older adults during the COVID-19 pandemic, to examine the association of individual precautionary behavior with older adults’ depression levels, and to identify the moderating role of socioeconomic indicators in the aforementioned association. Five hundred and sixteen older adults were recruited from five cities of Hubei province in China. They were asked to complete an online questionnaire survey. Results showed that 30.8% of participants indicated a significant depressive symptom during the pandemic. Older adults’ depression levels differed significantly in marital status, living situation, education level, household income, subjective health status, and infected cases of acquaintances. Precautionary behavior change showed significant inverse associations with older adults’ depression levels, where household income moderated this relationship. This is the first study to investigate the characteristics, behavioral correlates, and moderators of depression among Chinese older adults during the COVID-19 pandemic. Research findings may provide new insights into interventions and policy-making on individual precautionary behavior and mental health among older adults for future pandemics.

## 1. Introduction

Since the beginning of December 2019, a novel coronavirus disease (COVID-19), which was first recognized in Wuhan, Hubei province of China, quickly spread globally, infecting millions of people [1]. In China, there were 91,388 confirmed cases and 4746 fatality cases recorded by 13 October 2020 [2,3]. As a serious pandemic, the COVID-19 imposed enormous burdens on the medical system and exerted catastrophic impacts on social economics [4]. For individuals, the epidemic not only led to the risk of death from the viral infection but also augmented the comorbidity of mental illnesses (e.g., depressive symptoms) [5,6]. As a vulnerable group that accounted for the highest percentage of deaths from COVID-19 (approximately 75%), older adults have indicated a high risk of mental problems during the pandemic [7,8]. With the ongoing COVID-19 pandemic, older adults were more likely to experience fear of becoming ill or dying. This was accompanied by feelings of helplessness and stigma [8,9]. These feelings might result in an increased risk of depressive symptoms imposing profound negative influences on the health and well-being of older adults [8,9]. Previous studies have indicated a high prevalence of depressive symptoms from 22.3% to 39.1% among older adults during the COVID-19 outbreak [10,11,12,13]. Given the lack of relevant research, this emphasizes an urgent need for addressing the characteristics, correlates, and moderators of depression among older adults during the COVID-19 [13].

Since there has been very limited success in vaccination prevention for COVID-19, individual precautionary actions, such as hand washing, facemask wearing, and social distancing play a crucial role in inhibiting the human-to-human transmission of COVID-19 [14,15,16]. In addition, recent studies have indicated a significantly positive impact of precautionary behaviors on lessening the depressive symptoms among non-infected adolescents, adult populations, and adults with mental illnesses during the outbreak of COVID-19 [5,17]. The findings may generate urgently needed insights into the association of precautionary behaviors with mental health in the general population. However, to the best of our knowledge, there are limited studies examining the impact of COVID-19 precautionary behaviors on depression in older adults.

Socioeconomic status (SES) indicators, including education level, employment status, and household income, have been shown to be important predictors for precautionary behaviors and depression levels in the general population, respectively [17,18,19,20,21,22]. For instance, evidence has indicated a significantly positive association of education level and household income with the engagement of COVID-19 precautionary behaviors [15,19]. In addition, low education levels, unemployment status, and low household income have been demonstrated to be significantly correlated with a higher level or an increased risk of depression in previous studies [18,20,23]. The SES indicators have been considered to moderate the impact of certain health behaviors (e.g., physical activity, smoking, and social activities) on depression levels [24,25,26,27], whereas the moderating effects of SES in the relationship between COVID-19 precautionary behaviors and depression among older adults is still unknown. This deserves further examination, as identifying the socioeconomic characteristics associated with older adults’ depression levels is important. The effects of SES indicators on the relationships between precautionary behaviors and depression will help to develop tailored approaches to tackle the depression problems of the elderly population during the COVID-19 outbreak and future pandemics. In addition, the effects of specific SES indicators may also provide practical policy implications and enable the efficiency and feasibility of potential policy interventions to help combat COVID-19 and future pandemics [28].

The current study aimed to (1) investigate the characteristics of depression among Chinese older adults during the COVID-19 pandemic; (2) examine the association between individual precautionary behavior and older adults’ depression levels; and (3) identify the role of SES indicators (education level, occupational status, and household income) in moderating the association between individual precautionary behavior and depression levels in Chinese older adults. We hypothesized that (1) older adult’s depression levels would differ significantly for demographic characteristics, such as age, gender, marital status, and socioeconomic indicators; (2) taking up more COVID-19 precautionary behavior would be significantly associated with lower depression levels in older adults; (3) certain SES indicators would significantly moderate the association between individual precautionary behavior and depression levels in Chinese older adults.

## 2. Materials and Methods

### 2.1. Study Design and Participants

This study used a cross-sectional design using a snowball sampling approach. The online survey was conducted from 15 June to 10 July 2020 (the lockdown had been withdrawn for around two months). We contacted 727 Chinese older adults from five cities (e.g., Wuhan, Xiaogan, Jingzhou, Shiyan, and Xiangyang) in the Hubei province of China. A total of 609 participants (609/727, 83.8% response rate) agreed to participate in the survey. All of the participants who were community-dwelling older adults (≥60 years) met the eligibility criteria, which included: (1) not having been infected with COVID-19; (2) not having any cognitive disorders or impairments; (3) having access to a mobile phone or laptop; and (4) having sufficient reading and listening skills in Chinese. For those older adults who had difficulties in mobile phone or laptop operation, their family members and friends were invited to assist them in completing the online survey. Finally, there were 516 eligible participants (516/727, 71.0%), including 299 females (57.9%) and 217 males (42.1%), aged from 60 to 89 years (mean = 67.55 years, SD = 6.60).

### 2.2. Procedure

To minimize face-to-face interaction as recommended by the Chinese government, the questionnaire survey was constructed and administered using an online survey platform in China, namely, SOJUMP (Changsha Ranxing Information Technology Co., Ltd., Changsha, China). Four older adults (two males and two females) were invited to complete a pilot test with the purpose of (1) improving the layout of the electronic questionnaires (e.g., using the large font and highlighting the key information), and (2) modifying the grammar and any typographical errors while ensuring the scale items were more understandable [29]. All recruitment posters and the hyperlink for the survey were disseminated via mobile short message service (SMS) and popular social media platforms in China (e.g., WeChat, Weibo, and QQ). There were three approaches used for recruiting participants. (1) Relying on the researchers’ social networks in five cities of Hubei province, the eligible family members, friends, and relatives of researchers were invited. The participants then encouraged their friends to join the survey. (2) Researchers contacted the directors of community neighborhood committees in Wuhan and Xiaogan, respectively, and sought their collaboration and support. Upon receiving the agreement of directors, researchers were permitted to enter into their community neighborhood WeChat groups to recruit eligible participants. (3) Researchers contacted officials who were in charge of the retirement in two universities in Wuhan. With the support of officials, a recruitment poster and survey hyperlink were delivered to their internal WeChat group, especially for retirement colleagues.

To increase the engagement of participation, each participant who completed the online survey was eligible for 30 RMB by electronic transfer as an incentive. Participants were asked to sign an informed consent form on the first page of the survey platform prior to completing the questionnaires. Ethical approval for conducting the study was obtained from the Research Ethics Committee of Hong Kong Baptist University (REC/19-20/0490).

### 2.3. Measurement

#### 2.3.1. Demographic Information

Demographic characteristics included age, gender (male/female), marital status (single/married/divorced or widowed), living situation (alone/with spouse, partners or children), medical history of chronic diseases (e.g., heart diseases, diabetes, cancer, respiratory illnesses, liver or kidney diseases), and three key indicators of socioeconomic status (SES) [18,20,23,28]. These included education level (primary school or below/middle or high school/college or above), occupational status (unemployed/pensioner or retired/part-time or full-time employment), and household income (below average/average/above average). Participants were also asked to report their body weight and height for the calculation of body mass index (BMI), using the formula “BMI = body weight (kg)/body height squared (m^2^)”. Based on previous literature, the BMI was categorized by four levels for Chinese people (underweight: BMI < 18.5; healthy weight: 18.5 ≤ BMI < 23; overweight: 23 ≤ BMI < 26; and obesity: BMI ≥ 26) [30].

#### 2.3.2. Covariates

The acquaintances’ disease status and subjective health status served as important covariates for older adult’s depression [31,32]. Participants were asked to report the infection situation of COVID-19 of their acquaintances (e.g., friends, family members, and neighbors), as well as their subjective health status (bad/satisfactory/excellent).

#### 2.3.3. Precautionary Behaviors

As recommended by the WHO, the precautionary behaviors for COVID-19 include hand washing, facemask wearing, and social distancing [14]. A six-item structured scale was used to measure the COVID-19 precautionary behaviors, with two items for each of the three behaviors. For example, the items for hand washing were “during the previous week, I adhered to washing my hands with soap and water or alcohol-based hand rub (for at least 20 s, on all surfaces of the hands)” followed by two situations including “(a) in a daily life situation, e.g., before eating, and (b) in a disease-related situation, e.g., after caring for the sick.” The items for facemask wearing were “during the previous week, I adhered to wearing a face mask properly” followed by two different situations including “(a) when visiting public places, and (b) when caring for the sick”. The items for the social distancing were “during the previous week, I adhered to social distancing” followed by two items including “(a) staying out of crowded places and avoiding mass gatherings when going outside of my home, and (b) keeping space (at least 1.5 m) between myself and other people who are coughing or sneezing.” All responses were indicated on a four-point Likert scale ranging from “1 = strongly disagree” to “4 = strongly agree”. A mean score of the total six items was calculated.

Participants were also invited to recall their precautionary behaviors before the outbreak of COVID-19 during seasonal influenza (i.e., past precautionary behaviors) using the same scale.

#### 2.3.4. Depression

Depression levels were measured using the Chinese version of the Epidemiologic Studies Short Depression Scale (CESD-10) [33,34]. The questions were asked: “In the past week, how often I feel”, followed by 10 items such as “I had trouble keeping my mind on what I was doing”. The responses were given on a four-point Likert scale, ranging from “0 = rarely (less than 1 day)” to “3 = for most of the time (5–7 days)”. The total score of the 10 items was calculated (≥10 indicating significant depressive symptoms) [35]. The CESD-10 has demonstrated satisfactory validity and internal consistency reliability among Chinese older adults (Cronbach’s alpha = 0.78–0.82) [33,34].

The package of questionnaires was delivered on the online survey platform, and all participants were asked to complete the survey using their mobile phones or laptops. The duration to complete the online survey was around 20 min.

### 2.4. Statistical Analysis

IBM SPSS 26.0 (Armonk, NY, USA) was used for data analyses. The diagnostic testing (e.g., outlier screening and distribution checking) was first conducted, and all data adhered to the normal distribution that the absolute values of skewness and kurtosis were <2. Descriptive statistics (e.g., mean, standard deviation, percentage) were used to describe baseline characteristics. The characteristics of depression were examined by independent T-test and one-way analysis of variance (ANOVA). Hierarchical linear regression models were used to explore the association of precautionary behaviors with depression. To control the influence of past precautionary behaviors, residualized change scores (RCS; calculated by conducting linear regression between past behaviors and current behaviors) were used [36]. In Model 1, the demographic variables were set as predictors for the depression level. Subsequently, two covariates were added to the regression analysis in Model 2. Finally, the RCS of COVID-19 precautionary behaviors was included as a predictor in Model 3, controlled for the significant demographics and covariates. The role of the SES indicators in moderating the behavior–depression association was examined using IBM SPSS Process (Model 1), and the 95% confidence intervals (CIs) of the standardized effects were estimated using the bias-corrected bootstrap approach (5000 resample). The 5% level (two-tailed) was taken as the statistical significance cutoff point.

## 3. Results

### 3.1. Sample Characteristics

The descriptive characteristics of the sample are presented in Table 1. The data of 516 eligible older adults were included in the analysis. Most participants were females (57.9%) and were aged between 60 and 69 years (68.6%). The majority of the older adults were married (83.7%) and lived with their spouse, partners, or children (90.7%). In terms of the medical histories, about half of the participants have suffered from chronic diseases (e.g., heart diseases, diabetes, or cancer). For SES indicators, only a small percentage of participants were illiterate or semi-illiterate (8.7%), the majority of participants were pensioners/retired (92.6%), and more than half of the sample indicated an average level of household income (57.9%). In terms of BMI, a considerable proportion of elderly participants were overweight or obese (52.1%). In addition, most participants perceived their health status as good or excellent (52.7%), and only 9.7% of participants reported that their family members, friends, or neighbors had been infected with the COVID-19. According to the cutoff point for depression (CESD-10 ≥ 10) [35], 30.8% of the participants indicated significant depressive symptoms during the outbreak of COVID-19.

### 3.2. Characteristics of Depression

As shown in Table 2, older adult’s depression differed significantly for different characteristics. There were no significant differences in depression levels for gender, medical history of chronic diseases, occupational status, and BMI intervals (*p* = 0.10–0.95). The results indicated that older adults who were divorced/widowed and lived alone showed significantly higher depression levels than those who were married (*p* < 0.001) and lived with their spouse, partners, and children (*p* = 0.035). The depression level was significantly lower for participants who had higher educational levels (*p* = 0.001) and higher household income (*p* < 0.001) relative to those with poorer socioeconomic status. In addition, older adults who perceived their health status as bad and poor (*p* < 0.001) and who had acquaintances infected with COVID-19 (*p* = 0.003) indicated significantly higher depression levels than those in the other categories.

### 3.3. Association of Individual Precautionary Behaviors with Depression

Based on the characteristics of depression, all demographic variables that have shown significant differences in the depression levels were included as predictors in the hierarchical linear regression models [31]. Dummy variables were generated for all polynomial predictors. Results revealed that two demographic variables were significant predictors for older adult’s depression levels, including education level and household income, which aggregately accounted for 7% of the variance in the depression level (*p* < 0.001). In terms of the covariates, both subjective health status and infected cases of participants’ acquaintances significantly predicted the depression level among participants, coupled with the demographics contributing to the explanation for 12 % of the variance in the depression levels (*p* < 0.001). After controlling for the demographic factors and covariates, changes in COVID-19 precautionary behaviors significantly predicted the depression of older adults (β = −0.18, 95%CI = −1.24 to −0.62, *p* < 0.001), contributing to a significant improvement in the variance explanation (ΔR^2^ = 0.03, *p* < 0.001). The total model accounted for 15% of the variance in depression level (*p* < 0.001). Details of multiple linear regression analyses are shown in Table 3.

### 3.4. Moderating Effect of Socioeconomic Status

**Figure 1:**
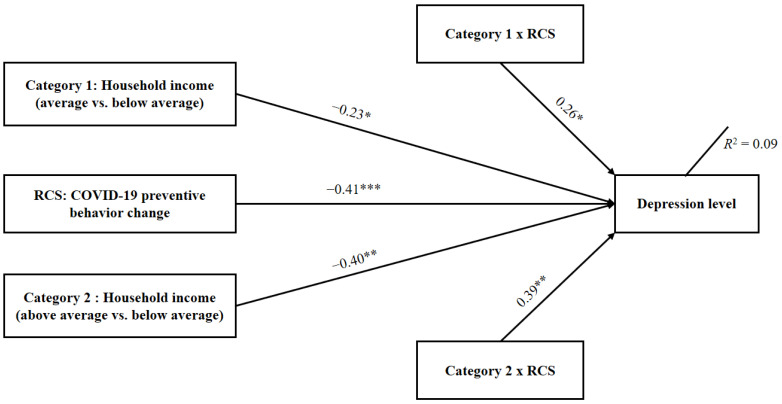
Moderation effect of household income on behavior-depression association (*n* = 516). RCS = Residualized change score; * *p* < 0.05; ** *p* < 0.01; *** *p* < 0.001.

**Figure 2:**
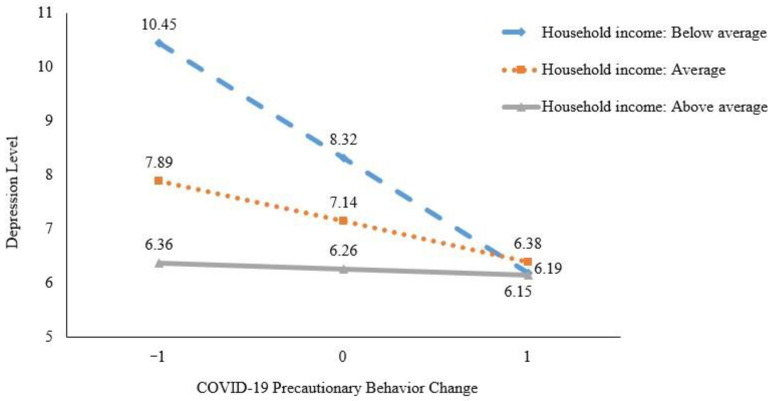
Plot of simple slopes showing the association between COVID-19 precautionary behavior change and depression level at different categories of household income.

As the occupational status was not significantly associated with depression in our previous examination (*r* = −0.04, *p* = 0.32), only education level and household income were included in the moderation analysis. The interaction of socioeconomic variables with precautionary behaviors was first examined and the results showed that education level was not significantly related with precautionary behaviors for predicting older adult’s depression (β_1_ = 0.12, *t*_510_ = 0.78, *p* = 0.44, 95%CI = −0.93 to 2.14; β_2_ = 0.04, *t*_510_ = 0.23, *p* = 0.82, 95%CI = −1.42 to 1.79). For the household income, a significant moderation effect was identified in the analysis (See Figure 1). Results indicated a significant interaction between household income (average vs. below average) and precautionary behaviors (β_1_ = 0.26, *t*_510_ = 2.53, *p* = 0.012, 95%CI = 0.31 to 2.44), as well as between household income (above average vs. below average) and precautionary behaviors (β_2_ = 0.39, *t*_510_ = 3.01, *p* = 0.003, 95%CI = 0.70 to 3.34). The interaction contributed to a significant change in the variance explanation (ΔR^2^ = 0.02, *p* = 0.007). The total moderation model accounted for 9% of the variance in depression (*p* < 0.001). The descriptive plot of the moderating effects of household income on the relationship between COVID-19 precautionary behavior change and depression level among older adults is presented in Figure 2. For older adults with higher levels of household income, there was only a slight negative association between precautionary behavior change and depression level, whereas for those with average and lower levels of household income, prominent associations between behavior change and depression levels occurred.

## 4. Discussion

To the best of our knowledge, this is the first online cross-sectional study to explore the characteristics of depression, to examine the association between COVID-19 precautionary behaviors and depression levels, and to identify the role of SES in moderating the behavior–depression association among Chinese older adults during the COVID-19 pandemic. The findings from the study have fully supported the hypotheses. In particular, during the outbreak of COVID-19, older adults’ depression levels differed significantly in a series of characteristics, including marital status, living situation, SES indicators (education level and household income), as well as subjective health status and infected cases of acquaintances. After controlling for the demographic covariates, COVID-19 precautionary behaviors showed a significant inverse association with older adults’ depression levels. Of the three SES indicators, only household income significantly moderated the association between COVID-19 precautionary behaviors and depression levels among Chinese older adults.

In terms of the characteristics of depression, as suggested in previous studies, individuals who lack social support from families and friends showed significantly higher levels of depression than those with sufficient social support from families and friends [37,38,39]. Therefore, it is not surprising that in this study, older adults who have married and lived with their spouse, partners, or children indicated a prominent lower depression level. The findings also revealed that older adults who perceived their health status as poor and had acquaintances being infected showed a significantly higher level of depression. These findings are consistent with previous studies, where older adults with these characteristics may experience greater fear of being infected or dying themselves, leading to higher depression levels [32,37,38]. In line with previous evidence in Chinese adolescents and adults, the findings showed that older adults who had higher education levels and higher household income might be less influenced by the COVID-19 pandemic, indicating a comparative lower depression level [5,22]. The discrepancy with previous evidence occurred in the indicator of occupational status [23,24], where no significant difference was found in the current study. This may be attributed to the reason that the majority of our participants were retired older adults (92.6%).

In terms of the association of individual precautionary behavior towards COVID-19 with depression levels, our findings were consistent with previous studies among Chinese non-infected adolescent and adult populations [5], and with a recent study among Japanese adults with depressive symptoms [17]. Older adults who adopted more precautionary behaviors (e.g., hand washing, facemask wearing, and social distancing) were more likely to have lower depression levels during the COVID-19 epidemic. It is worth noting that the change in COVID-19 precautionary behaviors accounted for 3% of the variance in depression levels, while the SES indicators (education levels and household income) and covariates (infected cases of acquaintances, subjective health status), also played a critical role in predicting older adults’ depressive states. These findings emphasize the significance of promoting precautionary behaviors during the COVID-19 pandemic among older adults, as well as the importance of considering the socio-demographic characteristics when designing psychological interventions and making relevant policies to improve mental health outcomes among older adults.

In terms of the moderating effect of SES indicators on the behavior–depression association, household income was found to be a significant moderator. This result agrees with previous studies [25], which indicate that the economic dependency significantly interacted with social activity and depression among older adults (β = −0.16, SE = 0.01, *p* < 0.001) [25]. The findings of the current study support the moderating role of household income in the behavior–depression association, revealing that when we motivate older adults to take COVID-19 precautionary behaviors to reduce their depression levels, we need to especially focus on older adults who are at an economically disadvantaged level. From the government’s perspective, the findings indicate the importance and necessity of providing relief funding for low-income households to ease the stress of the pandemic. These findings also bear considerable implications for future preventive measures of epidemics among older adults.

This study has several limitations. First, given the urgency of the research needed on the COVID-19 pandemic and the limited resources available, we have to apply an online cross-sectional approach using snowball sampling, so the participants may vary in relation to the actual patterns of the general elderly population (e.g., in the illiterate or semi-illiterate samples). Moreover, all the variables were measured by self-reported scales, which might lead to recall bias, self-perception bias, and social desirability effects [22,40]. However, the bias has been found to be lower in anonymous online surveys than in telephone or face-to-face paper surveys [15,41]. In spite of online methodologies being an efficient means and cost-effective method to conduct surveys, we adopted several strategies to ensure that the online survey was easy-to-operate. However, many elderly participants were confronted with difficulties in the process of the survey (e.g., operational functionality, submission setting). Further actions are needed to make online surveys more user-friendly for elderly populations. Additionally, the demographic and behavioral factors identified in the present study only explained 15% of the variance of depression levels, so other factors need to be investigated in future studies. In addition, the depression levels did not significantly differ in gender, whereas other studies have found a prominent role for the gender variable in the psychological responses towards the pandemic [21,22]. This point deserves further investigation. Finally, the findings of the present study were obtained from a specific age group within a Chinese context; therefore, it is unclear whether these findings would be generalizable to other age groups and different cultural contexts. Notwithstanding the aforementioned limitations, this study provides invaluable information on the characteristics of depression, and the impact of COVID-19 precautionary behaviors when considering depression levels. The study also provides detail relating to the role of SES indicators in moderating the behavior–depression association among Chinese older adults during the COVID-19 pandemic. The research findings presented here could be used as a meaningful reference, adding knowledge and giving new insights into future research promoting precautionary behaviors and relationships between mental health and older adults during the COVID-19 outbreak and potential future pandemics.

## 5. Conclusions

The current study investigated the characteristics of depression between Chinese older adults during the COVID-19 pandemic. The study also examined the association between older adult’s individual precautionary behaviors and their depression levels, and identified the role of SES indicators in moderating the behavior–depression relationship. All of the study hypotheses were supported. The depression levels of older adults differed significantly for marital status, living situations, education levels, household income, subjective health status, and infected cases of acquaintances. The inverse association between precautionary behavior change and depression levels was also identified in the current study. Of the three SES indicators, only household income significantly moderated the impacts of COVID-19 precautionary behaviors on older adults’ depression levels. The research findings highlight the potential for embracing COVID-19 precautionary behaviors on mitigating depression levels among older adults. The findings also revealed the importance of considering socioeconomic disparities when promoting precautionary behaviors for mental health. These findings could be important in influencing relevant social policy decisions that target older adults.

## Figures and Tables

**Table 1:** Descriptive characteristics of the study sample (*n* = 516).

	*n* (%)
**Age (years)**, mean (SD): 67.55 (6.60)	
60–69	354 (68.6)
70–79	128 (24.8)
80 and above	34 (6.6)
**Gender**	
Male	217 (42.1)
Female	299 (57.9)
**Marital status**	
Single	14 (2.7)
Married	432 (83.7)
Divorced or widowed	70 (13.6)
**Living situation**	
Alone	48 (9.3)
With spouse/partners/Children	468 (90.7)
**Medical history of chronic diseases**	
Yes	262 (50.8)
No	254 (49.2)
**Education level**	
Primary school or below	45 (8.7)
Middle or high school	231 (44.8)
College or above	240 (46.5)
**Occupational status**	
Unemployed	22 (4.3)
Pensioner or retired	478 (92.6)
Part-time or full-time employment	16 (3.1)
**Household income**	
Below average	113 (21.9)
Average	299 (57.9)
Above average	104 (20.2)
**BMI (kg/m^2^)**, mean (SD): 23.06 (2.67)	
<18.5	19 (3.7)
18.5 ≤ BMI < 23	228 (44.2)
23 ≤ BMI < 26	206 (39.9)
≥26	63 (12.2)
**Subjective health status**	
Bad	48 (9.3)
Satisfactory	196 (38.0)
Excellent	272 (52.7)
**Infected cases of acquaintances**	
Yes	50 (9.7)
No	466 (90.3)
**Depression**, mean (SD): 7.34 (5.23)	
No depressive symptom	357 (69.2)
Have depressive symptoms	159 (30.8)
**Precautionary behaviors**	
Before the outbreak of COVID-19, mean (SD): 3.12 (0.67)	
During the outbreak of COVID-19, mean (SD): 3.61 (0.40)	

Note. SD = standard deviation.

**Table 2:** Characteristics of depression (*n* = 516).

Factors	Depression Mean (SD)	Significance
**Age span**		*F*_2, 513_ = 1.78, *p* = 0.17
60–69	7.06 (5.04)	
70–79	7.88 (5.45)	
80 and above	8.29 (6.08)	
**Gender**		*t*_514_ = −0.06, *p* = 0.95
Male	7.33 (5.43)	
Female	7.35 (5.09)	
**Marital status**		*F*_2, 513_ = 7.87, *p* < 0.001
Single	9.07 (5.80)	
Married	6.96 (5.07)	
Divorced or widowed	9.57 (5.56)	
**Living situation**		*t*_514_ = 2.11, *p* = 0.035
Alone	8.85 (5.34)	
With spouse/partners/Children	7.19 (5.20)	
**Medical history of chronic diseases**		*t*_514_ = 1.66, *p* = 0.10
Yes	7.72 (5.26)	
No	6.96 (5.17)	
**Education level**		*F*_2, 513_ = 7.32, *p* = 0.001
Primary school or below	10.07 (5.94)	
Middle or high school	7.12 (5.02)	
College or above	6.86 (5.13)	
**Occupational status**		*F*_2, 513_ = 1.24, *p* = 0.29
Unemployed	9.01 (6.18)	
Pensioner or retired	7.29 (5.22)	
Part-time or full-time employment	6.75 (3.73)	
**Household income**		*F*_2, 513_ = 9.09, *p* < 0.001
Below average	9.06 (5.72)	
Average	7.08 (5.07)	
Above average	6.24 (4.70)	
**Body mass index (BMI) intervals**	23.06 (2.67)	*F*_3, 512_ = 0.62, *p* = 0.60
BMI < 18.5 kg/m^2^	6.37 (4.70)	
18.5 kg/m^2^ ≤ BMI < 23 kg/m^2^	7.62 (5.60)	
23 kg/m^2^ ≤ BMI < 26 kg/m^2^	7.08 (4.88)	
BMI ≥ 26 kg/m^2^	7.48 (5.10)	
**Subjective health status**		*F*_2, 513_ = 17.25, *p* < 0.001
Bad	10.44 (6.01)	
Satisfactory	8.09 (5.32)	
Excellent	6.26 (4.69)	
**Infected cases of acquaintances**		*t*_514_ = 2.95, *p* = 0.003
Yes	9.40 (6.18)	
No	7.12 (5.07)	

Note. SD = standard deviation.

**Table 3:** Results of hierarchical linear regression models (*n* = 516).

Predictors of Depression	Model 1			Model 2			Model 3		
	β	95% CI	*p*	β	95% CI	*p*	β	95% CI	*p*
**Marital status**									
Single (reference group)	N/A	N/A	N/A	N/A	N/A	N/A	N/A	N/A	N/A
Married	−0.08	−3.08, 0.80	0.25	−0.05	−2.60, 1.18	0.46	−0.03	−2.28, 1.45	0.66
Divorces or widowed	0.04	−1.41, 2.77	0.53	0.05	−1.24, 2.84	0.44	0.07	−1.01, 3.00	0.33
**Living situation**									
Alone (reference group)	N/A	N/A	N/A	N/A	N/A	N/A	N/A	N/A	N/A
With spouse/partners/children	<0.01	−0.58, 0.65	0.91	−0.01	−0.66, 0.53	0.83	0.01	−0.50, 0.68	0.76
**Education level**									
Primary school or below (reference group)	N/A	N/A	N/A	N/A	N/A	N/A	N/A	N/A	N/A
Middle or high school	−0.18	−3.06, −0.65	0.003	−0.17	−2.95, −0.60	0.003	−00.16	−2.81, −0.49	0.005
College or above	−0.19	−3.18, −0.75	0.002	−0.19	−3.17, −0.79	0.001	−0.16	−2.80, −0.45	0.007
**Household income**									
Below average (reference group)	N/A	N/A	N/A	N/A	N/A	N/A	N/A	N/A	N/A
Average	−0.15	−3.28, −1.27	<0.001	−0.11	−1.98, −0.40	0.003	−0.08	−1.66, −0.10	0.027
Above average	−0.18	−3.28, −1.27	<0.001	−0.13	−2.65, −0.66	0.001	−0.10	−2.33, −0.36	0.008
**Subjective health status**									
Bad (reference group)	N/A	N/A	N/A	N/A	N/A	N/A	N/A	N/A	N/A
Satisfactory	N/A	N/A	N/A	−0.16	−2.87, −0.63	0.002	−0.16	−2.85, −0.64	0.002
Excellent	N/A	N/A	N/A	−0.33	−4.53, −2.33	<0.001	−0.33	−4.55, −2.39	<0.001
**Infected cases of acquaintances**									
Yes (reference group)	N/A	N/A	N/A	N/A	N/A	N/A	N/A	N/A	N/A
No	N/A	N/A	N/A	−0.10	−1.44, −0.40	0.001	−0.11	−1.47, −0.45	<0.001
**Precautionary behaviors ^a^**	N/A	N/A	N/A	N/A	N/A	N/A	−0.18	−1.24, −0.62	<0.001
*R* ^2^	0.07, *p* < 0.001			0.12, *p* < 0.001			0.15, *p* < 0.001		
Δ*R*^2^	N/A			0.05, *p* < 0.001			0.03, *p* < 0.001		

Note. ^a^ Residualized change scores were used for the calculation.
